# Comparison of two psycho-educational family group interventions for improving psycho-social outcomes in persons with spinal cord injury and their caregivers: a randomized-controlled trial of multi-family group intervention versus an active education control condition

**DOI:** 10.1186/s40359-016-0145-0

**Published:** 2016-07-26

**Authors:** Dennis G. Dyck, Douglas L. Weeks, Sarah Gross, Crystal Lederhos Smith, Hilary A. Lott, Aimee J. Wallace, Sonya M. Wood

**Affiliations:** 1 Department of Psychology, Washington State University Spokane, 412 E. Spokane Falls Blvd., Spokane, WA 99202 USA; 2 St. Luke’s Rehabilitation Institute, 711 S. Cowley St., Spokane, WA 99202 USA; 3 Department of Biomedical Sciences, Elson S. Floyd College of Medicine, Washington State University, Spokane, WA 99202 USA; 4 College of Nursing, Washington State University Spokane, 412 E. Spokane Falls Blvd., Spokane, WA 99202 USA

**Keywords:** Study protocol, Spinal cord injury, Caregiver, Psycho-educational intervention, Multi-family group treatment

## Abstract

**Background:**

Over 12,000 individuals suffer a spinal cord injury (SCI) annually in the United States, necessitating long-term, complex adjustments and responsibilities for patients and their caregivers. Despite growing evidence that family education and support improves the management of chronic conditions for care recipients as well as caregiver outcomes, few systematic efforts have been made to involve caregivers in psycho-educational interventions for SCI. As a result, a serious gap exists in accumulated knowledge regarding effective, family-based treatment strategies for improving outcomes for individuals with SCI and their caregivers. The proposed research aims to fill this gap by evaluating the efficacy of a structured adaptation of an evidence-based psychosocial group treatment called Multi-Family Group (MFG) intervention. The objective of this study is to test, in a randomized-controlled design, an MFG intervention for the treatment of individuals with SCI and their primary caregivers. Our central hypothesis is that by providing support in an MFG format, we will improve coping skills of persons with SCI and their caregivers as well as supportive strategies employed by caregivers.

**Methods:**

We will recruit 32 individuals with SCI who have been discharged from inpatient rehabilitation within the previous 3 years and their primary caregivers. Patient/caregiver pairs will be randomized to the MFG intervention or an active SCI education control (SCIEC) condition in a two-armed randomized trial design. Participants will be assessed pre- and post-program and 6 months post-program. Intent to treat analyses will test two a priori hypotheses: (1) MFG-SCI will be superior to SCIEC for SCI patient activation, health status, and emotion regulation, caregiver burden and health status, and relationship functioning, and (2) MFG will be more effective for individuals with SCI and their caregivers when the person with SCI is within 18 months of discharge from inpatient rehabilitation compared to when the person is between 19 and 36 months post discharge.

**Discussion:**

Support for our hypotheses will indicate that MFG-SCI is superior to specific education for assisting patients and their caregivers in the management of difficult, long-term, life adjustments in the months and years after SCI, with increased efficacy closer in time to the injury.

**Trial registration:**

ClinicalTrials.gov NCT02161913. Registered 10 June 2014.

## Background

In the United States, an estimated 276,000 individuals currently live with spinal cord injuries (SCIs) [1]. The vast majority of SCIs are the result of unexpected events (i.e., vehicular accidents, falls, acts of violence, and accidents that occur during sports and recreation) that immediately, dramatically, and permanently change the lives of those who experience them [2]. Over half of the individuals who experience SCI are healthy [3] and employed [4] at the time of their injuries. The vast array of loss that ensues is life-shattering and includes loss of mobility, loss of functional abilities, changes in sensory and autonomic functioning, multisystem impairment, risk of serious secondary complications, and decrease in life expectancy [4, 5]. Not only are those who experience SCI largely young–between the ages of 15 and 35 years–they are most frequently single young men [3, 4, 6] who find themselves unable to maintain employment [4] as a result of related disability [7] and suddenly dependent upon others for their care [8]. Physical limitations related to their injuries also limit their social interaction and interfere with their ability to perform social roles [9, 10]. Each of these stressors contributes to extremely high levels of psychological distress and morbidity [8], including increased risk for substance abuse [11], decreased life satisfaction [12], decreased social integration, and increased loneliness [9, 10].

The vast majority of those who experience SCI are sent home after discharge from the hospital [4]. Family members often find themselves in the role of caregivers, serving as advisors, educators, advocates, and prevention / management specialists concerning health complications, as well as providing financial support [5]. While such support has been described as indispensable for individuals with SCI [5], it significantly strains family members in these positions [13–15]. Caregivers often report chronic health problems, feelings of frustration, isolation, guilt, and resentment toward their injured family member [16]; spouses of individuals with SCI have been found to self-report even higher levels of distress than with the person with SCI [17]. There is an obvious need for psychologically based interventions aimed at improving the health status and quality of life for individuals with SCI and those who care for them [8].

Decades of research and meta-analytic reviews have demonstrated that the education and involvement of caregivers in the treatment of persons with other chronic conditions (e.g., severe mental illness) results in improved rehabilitative and community outcomes relative to those receiving individual treatment or treatment as usual [18–20]. When used with schizophrenia and traumatic brain injury (TBI), one such approach that engages care recipient and caregiver in co-treatment, termed Multi-Family Group (MFG) psycho-education, has been found to improve the management of these conditions and improve caregiver outcomes as well [21–24]. However, few systematic efforts have been made to involve caregivers in psycho-educational interventions designed to improve adjustment to SCI. As a result, a serious gap exists in accumulated knowledge regarding effective, family-based treatment strategies for improving outcomes for individuals with SCI and their caregivers. This study aims to fill this gap by evaluating the efficacy of a structured adaptation of an evidence-based group treatment for SCI. This psychosocial intervention, MFG for SCI (MFG-SCI), builds on earlier work by Dyck and colleagues [21].

The purpose of this study is to rigorously test the previously developed MFG intervention for persons with SCI and their primary caregivers. We will recruit individuals with SCI who have been discharged from inpatient rehabilitation within the previous 3 years and their primary caregivers. The MFG intervention will be compared to an active control condition (SCIEC) in a two-armed randomized trial design. We will test the following hypotheses in the proposed study: 1) The MFG intervention relative to the SCIEC condition will result in greater improvement in quality of life, health activation, and health status and psychological functioning among individuals with SCI; it will also result in reduced caregiver burden among informal caregivers, as well as improved relationship quality. 2) The MFG intervention will be shown to be more effective for individuals with SCI and their caregivers when the patient is within 18 months of discharge from inpatient rehabilitation (e.g., sub-acute phase of adjustment to injury), compared to when the patient is between 19 and 36 months post discharge from inpatient rehabilitation (e.g., chronic phase of adjustment to injury).

## Methods and design

We will randomize 32 outpatients with SCI and their caregivers to a 9 month MFG intervention (MFG-SCI, *n* = 16 pairs) or to an SCI education control condition (SCIEC, *n* = 16 pairs) of the same duration. Over time, each condition will have 4 to 5 cohorts of 3 to 5 couple pairs. Participants will receive the different treatments at a single medical rehabilitation hospital. All participants will provide written informed consent prior to participation. The SCIEC condition will be based on the 4th Edition of the book titled: *Yes, you can: A guide to self-care for persons with spinal cord injury* [25]. The content will be taken from four sections of the book and will be covered in 16 bi-monthly sessions: 1. How SCI affects your body; 2. Maximizing your function; 3. Coping and Living with SCI; and 4. Staying healthy with SCI. The study will employ a two-arm randomized intervention-control design with repeated measures of outcomes across time. Depending on characteristics of individuals with SCI who consent to participate, we will stratify participants on time since injury (≤18 months post discharge vs. 19–36 months) and location/severity of the SCI (American Spinal Injury Association [ASIA] Classification system) and randomize to the two conditions, rigorously assessing outcomes of those with SCI and their caregivers. The SCIEC intervention will provide SCI management information in a didactic format, but it will not include the group problem-solving or structured social support afforded by MFG over the same 9-month period. Each of the clinicians will be provided a manual for didactic components of the intervention; in addition, they will receive bi-weekly supervision by the PI. For all participants, we will continue to measure outcomes after completion of the 9-month intervention period in order to assess durability of outcomes.

Eligibility criteria for individuals with SCI will include: having an SCI (quadriplegia or paraplegia) due to an acquired injury with complete or incomplete lesion as defined by ASIA; discharge from inpatient rehabilitation within the previous 3 years; being age 16 years or older; having a mobility impairment as the result of the SCI; living in the community in a non-group setting after injury; planning to remain in the geographic area for at least 12 months; and competency in English. Dyads will be excluded if the primary caregiver or person with SCI has a terminal illness with life expectancy of less than 12 months; is in active treatment for cancer; is blind or deaf; or has a moderate to severe cognitive impairment (defined at screening as a score on the Short Portable Mental Status Questionnaire > 4 errors).

Eligibility criteria for primary caregivers will include provision of instrumental or emotional support for a spouse, relative, partner, or friend with SCI; having regular contact with the individual with SCI (at least a minimum of 2 h face-to-face contact per week); living with or near the individual with SCI; being age 18 or over; having a telephone; planning to remain in the geographic area for at least 12 months; and competency in English.

### Outcome measures

Individuals with SCI and their caregivers will be assessed pre- and post-treatment and 6 months post-treatment (see Table 1). Outcomes will be assessed with psychometrically validated quantitative measures for persons with SCI and caregivers. We will also measure the quality of the dyad’s relationship. Qualitative focus groups will be conducted with all participants at the end of the treatment periods to uncover participant perceptions of overall group experience, how useful it was, suggestions for improvement, and information and/or coping skills they learned to support rehabilitation or care giving.

**Table 1:** SCI and caregiver assessments and measurement schedule

		Pre-treatment	Post-treatment	6-months
SCI Patient Assessments				
Patient Activation				
	**Patient Activation Measure (PAM)**	X	X	X
Emotion Regulation/Interpersonal Skills				
	AX Scale (anger expression)	X	X	X
	Duke Social Support Index (use of supports)	X	X	X
Mental Health/Health Behavior				
	CES-D-10 (depression)^a^	X	X	X
Neuropsychological				
	Mini-Mental Status Examination	X		
Caregiver Assessments				
Caregiver Burden/Health				
	**Caregiver Burden Inventory**	X	X	X
	**CES-D-10 (depression)** ^**a**^	X	X	X
Dyad Functioning (administered to patient and care partner)				
	F-COPES	X	X	X

Bolded measure indicates primary outcome measure

^a^covariate in analyses

### SCI patient assessments

The Patient Activation Measure (PAM) [26] will be used to assess our primary quality of life outcome: health/patient activation. The PAM will measure the degree of individual’s knowledge, confidence, and skill to participate in self-management [26]. A higher degree of patient activation has been associated with better health outcomes for adults with chronic conditions [27–30]. Two measures will be used to assess our secondary outcomes of anger-expression and social support (i.e., change in the person with SCI’s use of adaptive social behaviors needed for effectively coping with SCI): the Anger Expression Scale (AXS) [31] will measure anger management including anger-in (suppression of angry feelings), anger-out (expression of anger towards property or people) and anger control (the frequency of attempts to control expressions of anger). The Abbreviated Duke Social Support Index (ADSSI) [32] will be used to measure both subjective support and social network interactions. We will also evaluate the presence and severity of depressive symptoms as these may influence benefits derived from treatment. The 10-item form of the widely-used, reliable Center for Epidemiologic Study of Depression (CESD-10) [33] will assess level of current depressive symptoms.

### Caregiver assessments

The Caregiver Burden Inventory (CBI) [34] will evaluate caregiver burden in four areas: physical, social, emotional and time dependence burden. The CESD-10 will be used to evaluate depressive symptoms during the past 6 months.

### Dyad functioning

The Family Crisis Oriented Personal Evaluation Scales (F-COPES) [35] assesses family-level coping including use of social/spiritual support, reframing negative events, and mobilizing the family to acquire/accept help. The F-COPES has been widely used with internal consistency reliability ranging from .82 to .89 [36]. The F-COPES will allow us evaluate, on an exploratory basis, whether improved dyad functioning is mediated by improved coping and will be administered to both dyad members.

### Overview of the MFG intervention

MFG-SCI uses a structured problem-solving and skills training approach to provide individuals with SCI and their caregivers with tools and information to improve coping and help family members to reconnect through positive behavioral exchanges. MFG educators are health professionals with experience in management of SCI, such as physical therapists, occupational therapists, and psychologists. MFG-SCI consists of three sequential phases: (1) a “Joining” in which MFG educators meet with each individual dyad for 2–3 sessions to allow participants to become acquainted with the educators, evaluate ongoing problems, and define treatment goals; (2) a 2-session Educational Workshop which provides information about SCI to all persons with SCI and their caregivers; (3) bi-monthly multifamily group meetings for 6 months (12 sessions) which provide a structured format for building problem-solving and communication skills while receiving social support. These 12 sessions will be divided into three 4-session phases: SCI management and self-care, coping, living with SCI, and staying healthy after SCI. Through instilling a systematic approach to solving everyday problems related to SCI challenges, MFG aims to reduce emotional distress and improve skills and supports through enlisting the caregiver’s practical and emotional support for the person with SCI.

The educator joining with each couple also leads the group. Although the structure and contents of the MFG Joining and Workshop are provided in the relevant sections of the treatment, the focus here is on describing the structure of the MFG problem-solving sessions. The group sessions consist of 3 components: (1) A brief (15-min) period for socialization, unwinding and “small talk”; (2) after 15 min, the educator starts the “Go Round” in which each couple relates briefly how the past 2 weeks have gone for them, including follow-up on homework or problem-solving recommendations. The educators take this opportunity to amend plans which have not been successful, offering a modification of the original or an alternative solution. Based on the Go Round, a problem or goal is selected for the current week’s group exercise. Thirty-five minutes are allotted to the Go Round, including problem selection. (3) The educators then lead the group in formal problem solving for approximately 35 min, using a six step process based on brainstorming methods from organizational and business practices.

- Step 1. Define the problem or goal (MFG members & educators);
- Step 2. List all possible solutions (MFG members);
- Step 3. Discuss advantages and disadvantages of each in turn (MFG members & educators);
- Step 4. Choose the solution that best fits the situation (MFG members);
- Step 5. Plan how to carry out this solution (Educators);
- Step 6. Review implementation (Educators).

The proceedings will be recorded on a whiteboard, to facilitate group participation and record the results. After the problem-solving exercise, 5 min are reserved for a wind-down before ending. This treatment approach differs from those that deliver information or develop skills in a planned sequence. Instead, problems are addressed as they occur in the course of participants’ daily lives. Solutions to emergent or continuing problems are generated by the group and/or by the educators, drawing on their knowledge of general problem-solving and compensatory strategies keyed to specific problems (e.g. pain, bladder management, pressure sores, needed home modifications), using the educators as consultants. The solutions are then implemented as homework, and reviewed during the next session. This approach has the advantage of ecological validity, a key aspect of rehabilitation interventions often lacking in more formulaic interventions [37].

### Comparison condition: SCIEC

The SCIEC condition is a 16-session, highly structured educational intervention that provides information on how SCI affects the body; methods for maximizing function, coping, and living with SCI; and staying healthy with SCI. It also includes general guidelines for improving health behavior. The content for these areas is based on a highly recognized self-care guide for persons with SCI [25]. Areas of focus in MFG-SCI, such as coping with SCI problems or dyad relationship and family readjustment issues, are explicitly not addressed in SCIEC. Each SCIEC session follows the same structure, beginning with a presentation of the objectives for the current session and a brief review of material from the previous session before introducing the session’s topic and presenting information on one or two key problem areas. In order to limit opportunities for group interaction and development of group cohesion, SCIEC utilizes a traditional didactic model with information delivered by the educator in a classroom or lecture setting (where all chairs face the educator). In addition, the information provided is general and broad-based, rather than focused on individual participants’ concerns. To avoid overlap with MFG problem-solving skills training, individual health problems will not be discussed. Instead, participants will be referred to their provider or supplied with a referral as needed. By contrast, MFG-SCI is designed to foster group support by delivering skills training in a round-table setting where all group members are encouraged to join in problem-solving exercises. Furthermore, the MFG educators’ approach is collaborative, and the materials are drawn from the everyday problems brought in by group members. Consistent with an educational model, handouts summarizing session material are provided in SCIEC; whereas in MFG-SCI homework is assigned as an integral feature of skills training and rehearsal and repetition are critical components of skills acquisition.

Table 2 shows the key structural-conceptual differences between conditions, while Table 3 summarizes the overall structure of the two conditions, including the different phases, components, and basic material delivered in each intervention. Table 3 also demonstrates that the two conditions are identical in number of sessions, but differ in treatment strategies (skills training vs. general education without reference to or problem-solving about participants’ individual health concerns/behavior).

**Table 2:** Comparison of MFG-SCI and control treatment (SCIEC)

Treatment Component	MFG-SCI	SCIEC
Therapeutic Strategy	Skills training, problem solving, support	Information only
Contents	SCI effects on the body, maximizing function, coping, living and staying healthy with SCI	SCI effects on the body, maximizing function, coping, living and staying healthy with SCI
Target Group	Persons with SCI and caregivers	All persons with SCI and caregivers
Use of Group Dynamics/ Cohesion	*Social support promoted*: Entire group participates in problem-solving for each dyad and gives support and encouragement	*Social support minimized:* Individual health issues not discussed, education is general, group interaction minimized
Therapeutic Stance	Educator stance is collaborative	Educator stance is didactic
Room Set-up	Round table	Lecture style (all chairs face forward)
Source of Material	Drawn from everyday problems brought in by group members	Supplied by educator
Homework	Assigned and reviewed at the start of the following session	Handouts but not homework provided

**Table 3:** Comparison of MFG-SCI and control treatment (SCIEC)

Treatment Component	MFG-SCI	# sessions	SCIEC	# sessions
Joining	*Dyad-tailored Education*: ^a^ SWOT analysis, SCI problems identified and corrected.	2(3) ^b^	*Standard Dyad Intake:* History of person with SCI and caregiver focusing on current health, skin care, bladder management, bowel management. No skills training, interventions, or formulation of management problems and needed adjustments.	2(3) ^b^
	*Formulation* of management problems and coping. Recommend one or more strategies and adjustments (individual and dyad).			
Group Introductory Sessions	*Educational Workshop:* ASIA classification, clinical syndromes, rehab therapy, medications, health lifestyle, the family and adjustment, family guidelines. Structure and function of multifamily group, how it can help.	2	*SCIEC Education Introduction:* Structure and rationale for intervention. Rules of conduct. Overview of topics to be covered.	1
Ongoing Group Sessions	*Problem-solving & Skills Training Sessions:* Problem-solving designed to address specific problems associated with SCI. Compensatory strategies for SCI problems, planning ahead.	12	*SCIEC Education:* General information provided to promote healthy living in areas relevant for persons with SCI and caregivers (bladder/bowel management, nutrition, use of alcohol, drugs, safe exercise).	13
			Personal health concerns not discussed; however, discuss referral to provider.	
Total		16		16

^a^In addition to basic intake

^b^The default is 2 sessions, an optional 3^rd^ session may be used to maintain contact with group members recruited early, or where the dyads are uncertain about continued participation

### General analytic approach

Preliminary analyses will include inspection of descriptive statistics and features of the data to determine whether data transformations for non-normal data are necessary. We will initially test whether baseline characteristics of the study population (e.g., age, sex, race, neurological level, extent of lesion) and other variables known to be related to the primary outcomes are comparable between the two treatment groups using independent t-tests or Wilcoxon rank-sum tests for continuous variables, and chi-square or Fisher’s exact tests for categorical variables. Groups will be considered imbalanced on variables that differ at *p* < .10, and all such imbalanced baseline prognostic factors will be included in primary analyses as covariates. We intend to measure the primary and secondary outcomes described in Table 1 at three time points for each participant (SCI patient and caregiver). Because repeated measurements on individual subjects tend to be correlated and, in some cases, the number and intervals of time between observations may vary among subjects, we will analyze each outcome measure with a general linear mixed model (GLMM) for longitudinal (repeated measures) data by modeling for 3 independent variables: Group (MFG-SCI vs. SCIEC), Time Since Inpatient Treatment (0–18 months vs. 19–36 months post-treatment), and Time of Assessment (pre-treatment, post-treatment, and 6-month follow-up) with covariates represented in the analyses as necessary, and individual subject variables will be simultaneously modeled as random effects. GLMM will account for dependence in repeated measures and accommodate correlated errors, unequal correlations among time points, unbalanced data resulting from missing data points, and unequal intervals between testing occasions. All principal analyses will be conducted based on the intention-to-treat principle in which any participant randomized to a treatment arm remains in it regardless of adherence to or completion of treatment. We will measure level of participation and conduct a sensitivity analysis that assesses the stability of the conclusions from the intention-to-treat analysis against an available-case analysis that considers only data from fully-adherent participants in a General Linear Model (GLM) repeated measures analysis of variance (RMANOVA). With multiple time points and variables representing outcomes at both the individual and caregiver levels, the planned analyses involve multiple comparisons, which increases likelihood that any single outcome will be found to be statistically significant based on chance alone. In order to minimize this risk, we have carefully selected a limited number of outcomes and clearly designated primary and secondary outcomes. Accordingly, we will also employ a more stringent observed type I error criterion (*p* = .01) than the typical .05 criterion in order to buffer against potential inflation of Type I errors due to multiple tests being performed in both the GLMM and RMANOVA.

### Tests of specific study hypotheses

Statistical analyses will test each of our a priori hypotheses that (1) MFG-SCI will be superior to SCIEC for SCI quality of life measures (patient activation, health status), and emotion regulation, caregiver burden and health status, and relationship functioning, and (2) MFG will be more effective for individuals with SCI and their caregivers when the person with SCI is within 18 months of discharge from inpatient rehabilitation compared to when the patient is between 19 and 36 months post discharge from inpatient rehabilitation. GLMM and RMANOVA will test main effects for Group, Time since Inpatient Treatment, and Time of Measurement, as well as the Group-by-Time of Measurement and Time since Inpatient Treatment-by-Time of Measurement interactions. GLMM will also be used to estimate effect sizes for each main effect.

### Power calculations

No studies comparing MFG to a control condition in an SCI population exist from which to estimate power. Therefore, we have used a recent trial that included Dr. Dyck as a co-investigator implementing MFG with survivors of TBI and their families [24]. Significant decreases were reported in TBI patient anger-expression, social support, and occupational activity, with caregivers reporting significantly decreased caregiver burden; all *p*’s < .05. Effect sizes across measures ranged from .6 to 1.0. The proposed study will be powered to determine whether the MFG-SCI is superior to the SCIEC control condition in 2 × 2 × 3, group-by-time since inpatient rehabilitation (0–18 months post-inpatient rehab vs. 19–36 months) by measurement time (baseline, post-treatment, 6-month follow-up) analyses of variance with repeated measures on the second factor. We have elected to estimate statistical power for the more conservative RMANOVA than for the more liberal GLMM procedure in order not to overestimate power. Given the lower bound effect size of .6, a 2-sided type I error rate of 0.01 [24], 1 degree-of-freedom for each between-groups comparison and 2 degrees-of-freedom for the repeated measures main effect, and an estimated correlation among repeated measures of 0.2, at a sample size per group of 16 (32 total dyads), the power to establish superiority of the intervention over the control condition is estimated to be 97 % across the primary and secondary endpoints for SCI patients and caregivers. If we were to lose four dyads to attrition, reducing number of dyads to 28 (14 per group), statistical power given the parameters above would be 95 %. Additionally, at a more conservative effect size of .5, at a sample size of 28, we would still have 87 % power to detect a main effect among groups. Thus, we have good power to detect a medium to large effect size even with a moderate degree of attrition. All power calculations were made with PASSv11.

## Discussion

There is currently a knowledge gap concerning how to best help individuals with SCI incorporate effective management strategies into their everyday lives to support coping and functional independence. There is also a paucity of individualized educational and support services for families living with the consequences of SCI. This project will address this gap by conducting a randomized, controlled study to evaluate the effectiveness of the MFG intervention tailored for persons with SCI and their caregivers. Group members with SCI and their caregivers will be provided information about how SCI affects the body and how to maximize adjustments and functioning. They will also learn guidelines for coping, living, and staying healthy after experiencing SCI. Participants will be taught self-care strategies related to SCI, given practice in solving problems related to SCI, and have the opportunity to exchange experiences and coping strategies with other care dyads over an 8 to 9 month period. While the content of the sessions will be guided by set topics, the problem-solving foci will also be informed by information provided by participants during the initial individual ‘joining’ sessions and throughout the MFG treatment. If study outcomes support our hypotheses showing superior efficacy for MFG-SCI vs. SCIEC, MFG implementation could potentially improve the quality of life for many persons with SCI and their caregivers.

## Abbreviations

ASIA, American Spinal Injury Association; GLMM, general linear mixed model; MFG, multi-family group; RMANOVA, repeated measures analysis of variance; SCI, spinal cord injury; SCIEC, spinal cord injury education control; TBI, traumatic brain injury

## References

[1] National Spinal Cord Injury Statistical Center (2015). Facts and figures at a glance.

[2] National Spinal Cord Injury Statistical Center (2015). Recent trends in causes of spinal cord injury.

[3] Lucke KT, Martinez H, Mendez TB, Arevalo-Flechas LC (2013). Resolving to go forward: the experience of Latino/Hispanic family caregivers. Qual Health Res.

[4] Facts and Figures at a Glance. The National Spinal Cord Injury Statistical Center Web site. https://www.nscisc.uab.edu/PublicDocuments/fact_figures_docs/Facts%202013.pdf. 2013. Accessed 3 Jun 2013.

[5] Guilcher SJT, Casciaro T, Lemieux-Charles L, Craven C, McColl MA, Jagial SB (2012). Social networks and secondary health conditions: the critical secondary team for individuals with spinal cord injury. J Spinal Cord Med.

[6] Burke DA, Linden RD, Zhang YP, Maiste AC, Shields CB (2001). Incidence rates and populations at risk for spinal cord injury: a regional study. Spinal Cord.

[7] The incidence and prevalence of spinal cord injury in Canada: overview and estimates based on current evidence. The Urban Future Web site. http://www.urbanfutures.com/spinal-injury. 2010. Accessed 3 Jun 2013.

[8] North NT (1999). The psychological effects if spinal cord injury: a review. Spinal Cord.

[9] Schulz R, Czaja SJ, Lustig A, Zdaniuk B, Martire LM, Perdomo D (2009). Improving the quality of life of caregivers of persons with spinal cord injury: a randomized clinical trial. Rehabil Psychol.

[10] Waite LJ, Hughes ME (1999). At risk on the cusp of old age: living arrangements and functional status among Black, White, and Hispanic adults. J Gerontol B Psychol Sci Soc Sci.

[11] Craig A, Tran Y, Middleton J (2009). Psychological morbidity and spinal cord injury: a systematic review. Spinal Cord.

[12] Post MWM, van Leeuwen CMC (2012). Psychosocial issues in spinal cord injury: a review. Spinal Cord.

[13] Post MW, Bloemen J, de Witte LP (2005). Burden of support for partners of persons with spinal cord injuries. Spinal Cord.

[14] Hughes ME, Waite LJ, LaPierre TA, Luo Y (2007). All in the family: the impact of caring for grandchildren on grandparents’ health. J Gerontol B Psychol Sci Soc Sci.

[15] Boschen KA, Tonack M, Gargaro J (2005). The impact of being a support provider to a person living in the community with a spinal cord injury. Rehabil Psychol.

[16] Kester BL, Rothblum ED, Lobato D, Milhous RL (1988). Spouse adjustment to spinal cord injury: long term medical and psychosocial factors. Rehabil Couns Bull.

[17] Weitzenkamp DA, Gerhart KA, Charlifue SW, Whiteneck GG, Savic G (1997). Spouses of spinal cord injury survivors: the added impact of care giving. Arch Phys Med Rehabil.

[18] Pilling S, Bebbington P, Kuipers E, Garety P, Geddes J (2002). Psychological treatments in schizophrenia: I. Meta-analysis of family intervention and cognitive behaviour therapy. Psychol Med.

[19] Mueser KT, Noordsy DL, Drake RE, Fox L (2003). Integrated treatment for dual disorders: a guide to effective practice.

[20] Glynn SM, Dixon LB, Cohen A, Murray-Swank A (2008). The family member provider outreach program. Psychiatr Serv.

[21] Rodgers ML, Strode AD, Norell DM, Short RA, Dyck DG, Becker B (2007). Adapting multiple-family group treatment for brain and spinal cord injury intervention development and preliminary outcomes. Am J Phys Med Rehabil.

[22] Dyck DG, Hendryx MS, Short RA, Voss WD, McFarlane WR (2002). Service use among patients with schizophrenia in psychoeducational multiple-family group treatment. Psychiatr Serv.

[23] Perlick DA, Straits-Troster K, Strauss JL (2013). Implementation of multifamily group treatment for veterans with traumatic brain injury. Psychiatr Serv.

[24] McFarlane WR (2002). Multiple family groups in the treatment of severe psychiatric disorders.

[25] Hammond MC, Burns SC (2009). Yes, you can!: A guide to self-care for persons with spinal cord injury.

[26] Hibbard JH, Stockard J, Mahoney ER, Tusler M (2004). Development of the Patient Activation Measure (PAM): conceptualizing and measuring activation in patients and consumers. Health Serv Res.

[27] Greene J, Hibbard JH (2012). Why does patient activation matter? An examination of the relationships between patient activation and health-related outcomes. J Gen Intern Med.

[28] Hibbard JH, Mahoney ER, Stock R, Tusler M (2007). Do increases in patient activation result in improved self-management behaviors?. Health Serv Res.

[29] Donald M, Ware RS, Ozolins IZ, Begum N, Crowther R, Bain C (2011). The role of patient activation in frequent attendance at primary care: a population-based study of people with chronic disease. Patient Educ Couns.

[30] Harvey L, Fowles JB, Xi M, Terry P (2012). When activation changes, what else changes? The relationship between change in patient activation measure (PAM) and employees’ health status and health behaviors. Patient Educ Couns.

[31] Knight RG, Chisholm BJ, Paulin JM, Waal-Manning HJ (1988). The Spielberger Anger Expression Scale: some psychometric data. Br J Clin Psychol.

[32] Koenig HG, Westlund RE, George LK, Hughes DC, Blazer DG, Hybels C (1993). Abbreviating the Duke Social Support Index for use in chronically ill elderly individuals. Psychosomatics.

[33] Irwin M, Artin KH, Oxman MN (1999). Screening for depression in the older adult: Criterion validity of the 10-item Center for Epidemiological Studies Depression Scale (CES-D). Arch Int Med.

[34] Novak M, Guest C (1989). Application of a multidimensional caregiver burden inventory. Gerontologist.

[35] Cockburn JT, Thomas FN, Cockburn OJ (1997). Solution-focused therapy and psychosocial adjustment to orthopedic rehabilitation in a work hardening program. J Occup Rehabil.

[36] McCubbin HI, Olson DH, Larsen AS, Corcoran K, Fischer J, Corcoran K, Fischer J (2000). Family crisis orientated personal evaluation scales [F-COPES]. Measures for clinical practice: a sourcebook.

[37] Cicerone K, Levin H, Malec J, Stuss D, Whyte J (2006). Cognitive rehabilitation interventions for executive function: Moving from bench to bedside in patients with traumatic brain injury. J Cogn Neurosci.

